# Kelvin probe force microscopy for local characterisation of active nanoelectronic devices

**DOI:** 10.3762/bjnano.6.225

**Published:** 2015-11-23

**Authors:** Tino Wagner, Hannes Beyer, Patrick Reissner, Philipp Mensch, Heike Riel, Bernd Gotsmann, Andreas Stemmer

**Affiliations:** 1 Nanotechnology Group, ETH Zürich, Säumerstrasse 4, 8803 Rüschlikon, Switzerland; 2 IBM Research — Zurich, Säumerstrasse 4, 8803 Rüschlikon, Switzerland

**Keywords:** capacitive crosstalk, frequency modulation, Kalman filter, Kelvin probe force microscopy, sidebands

## Abstract

Frequency modulated Kelvin probe force microscopy (FM-KFM) is the method of choice for high resolution measurements of local surface potentials, yet on coarse topographic structures most researchers revert to amplitude modulated lift-mode techniques for better stability. This approach inevitably translates into lower lateral resolution and pronounced capacitive averaging of the locally measured contact potential difference. Furthermore, local changes in the strength of the electrostatic interaction between tip and surface easily lead to topography crosstalk seen in the surface potential. To take full advantage of the superior resolution of FM-KFM while maintaining robust topography feedback and minimal crosstalk, we introduce a novel FM-KFM controller based on a Kalman filter and direct demodulation of sidebands. We discuss the origin of sidebands in FM-KFM irrespective of the cantilever quality factor and how direct sideband demodulation enables robust amplitude modulated topography feedback. Finally, we demonstrate our single-scan FM-KFM technique on an active nanoelectronic device consisting of a 70 nm diameter InAs nanowire contacted by a pair of 120 nm thick electrodes.

## Introduction

Device performance of current nanoelectronic devices, and even more so of potential future generations including nanowires or molecular junctions, critically depends on transport properties varying on a length scale of a few nanometres only in the active channel or at electrode interfaces. Methods for local electronic characterisation, providing accurate measurements with nanometre spatial resolution, are in very high demand, but have been lagging behind the technological requirements.

Kelvin probe force microscopy (KFM) is an established technique that allows for the mapping of local electrostatic potentials with an atomic force microscope (AFM) [1–3]. In contrast to electrostatic force microscopy (EFM), which measures merely the effect of electrostatic forces on the oscillation of the tip, a feedback loop nullifies the electric field by adjusting a bias voltage between tip and sample. Hence, Kelvin probe force microscopy is able to quantify the local contact potential difference (CPD), *U*_lcpd_, which contains contributions, e.g., from the difference in work function between the AFM tip and structures on the sample, dopants and trapped charges in the device, or voltages applied to electrodes.

For electronic devices on the nanoscale, KFM measurements provide a unique tool to shed light upon a variety of otherwise inaccessible properties. For example, with a constant current passing through a two-terminal device, the potential drop at the contacts directly relates to the contact resistance. To extract contact resistance through traditional four-point measurements becomes increasingly difficult for scaled devices, in which the contact length is comparable to the device length. Recently, KFM has been used to extract the surface state density and Schottky depletion region in semiconductor nanowires [4–5] or to determine the mean free path in carbon nanotubes [6]. KFM also allows one to determine intrinsic doping of two-dimensional crystals such as graphene [7–8], where surface potential and electronic properties depend on the number of layers.

KFM has found widespread use in both vacuum and ambient environments. Most commercial instruments for operation in air include a scan mode based on amplitude modulation KFM (AM-KFM). In this mode, the feedback loop nullifies the cantilever oscillation that is excited by a modulated electrostatic force. Hence, the KFM image is a map of voltages required to compensate the electrostatic force at every point of the scanned field. However, since cantilever and AFM tip are extended objects, this voltage does not necessarily correspond to the local contact potential difference, *U*_lcpd_, but represents a weighted average over the potentials present on the entire sample surface [9]. For AM-KFM, the weights are determined by the capacitance gradient, *C'*, between the probe and the sample. Due to the long range electrostatic force, even parts far from the surface, such as the cantilever beam, can account for a significant fraction of the signal, limiting the spatial resolution and accuracy of the measurement. Within nanoscale devices, for example, electrode potentials may completely overshadow the channel [10].

Known approaches to increase spatial resolution and accuracy of surface potential measurements include deconvolution techniques [11–12] or the use of slightly blunt tips supported by a cantilever of minimal surface area [9]. However, deconvolution techniques require a detailed model of the AFM tip to be accurate and usually neglect the sample topography [12], whereas blunt tips inevitably reduce topography resolution on three-dimensional structures.

**Figure 1:**
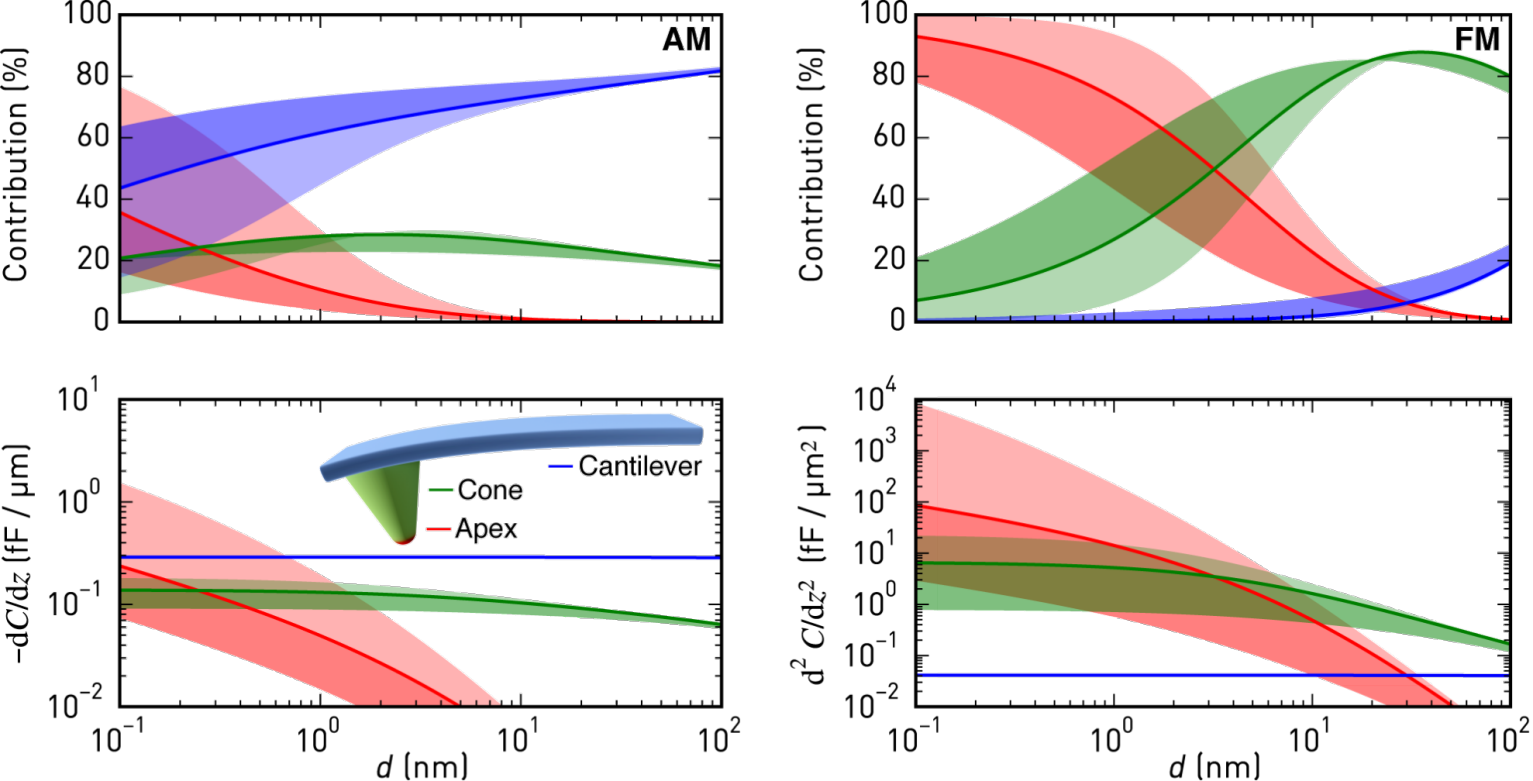
Contributions of apex, cone, and cantilever to the first (AM, left) and second (FM, right) order capacitance gradient as a function of distance for a mechanical oscillation amplitude of 5 nm (solid line) and an Olympus AC160 cantilever (*r*_tip_ = 5 nm, *H*_tip_ = 14 μm, θ = 17.5°, and *A*_lever_ = 160 μm × 40 μm). The light (dark) shaded regions indicate the range up to *A* = 0.1 nm (50 nm).

KFM measurements are further complicated by a strong dependence of the detected signal on the tip–sample distance. In the often employed lift-mode schemes, each line is scanned twice: first to acquire topography, and subsequently to retrace the scanned line at a small distance, Δ*z*, above the surface to perform KFM measurements. This enables tuning the ac modulation frequency for KFM to resonance to enhance the signal, and, at the same time, to reduce the contribution of van der Waals forces to the total force measured and compensated. The scan at elevated height, however, reduces lateral resolution and accuracy of the KFM data as we will detail below. To minimise such lateral averaging, single-scan methods are preferred, performing topography and KFM measurements simultaneously. An additional benefit of single-scan AFM and KFM is the inherent suppression of electrostatically induced topography artefacts present in non-compensated topography scans [13–14]. In AM-KFM, single-scan methods can be implemented taking advantage of multiple eigenmodes of the cantilever, using one mode for topography and another for KFM. Nevertheless, the averaging effect of the cantilever beam remains (see below in Figure 1).

An alternative approach typically applied in vacuum is based on frequency modulation [15]. To this end, the frequency of the cantilever is usually tracked by a phase-locked loop (PLL). Its output signal, the frequency shift Δ*f*, exhibits a frequency component at the electrostatic modulation frequency, which is nullified by the Kelvin feedback loop. Frequency modulated KFM (FM-KFM) [16–17] thus provides a map of potentials required to minimise the electrostatic force gradient, proportional to Δ*f* for small mechanical amplitudes, at every point during the scan. As a consequence, the contributions from different parts of the sample and the probe to the measured signal are weighted by the second-order capacitance gradient, *C''*, which effectively eliminates the averaging contribution of the cantilever beam as we explain in the following.

Figure 1 shows a model calculation using typical cantilever and interaction parameters, summarising how much tip apex, cone, and beam of an AFM cantilever probe contribute to the measured KFM signal in AM and FM operation. Shown are the percentages of the contributions and corresponding weighting factors *C'* and *C''* for AM and FM, respectively. To this end, we applied an analytic model of the electrostatic tip–sample interaction force [18] to the approximate geometry of a typically used cantilever (Olympus AC160), and we calculated *C'* and *C''* as a function of tip–sample separation for different oscillation amplitudes (see Supporting Information File 1 for details). While tip apex and cone clearly dominate the FM-KFM signal, opening the avenue to high resolution quantitative imaging, the cantilever beam at a distance of 14 μm dominates the AM-KFM signal even close to the sample, which is the main reason for the notoriously low lateral resolution and poor potential accuracy in this mode. When comparing AM and FM modes, one should note that in lift-mode AM-KFM the cantilever is not oscillating anymore when the electrostatic forces are nullified, whereas the mechanical oscillation remains in multifrequency AM-KFM and FM-KFM. Hence, for lift-mode the case *A* → 0 should be considered, whereas in single-scan modes the oscillation applied for tracking topography remains. For best sensitivity and minimal spatial averaging, AM and FM modes need to be operated very close to the surface.

While the FM-KFM approach is clearly superior in terms of signal composition, several issues complicate its use in practice. First, it is often performed together with frequency modulated topography feedback that employs a PLL to determine Δ*f*. The non-monotonous tip–sample interaction, by which Δ*f* can change its slope between net-attractive and net-repulsive forces, can complicate stable operation of the topography feedback and may ultimately render PLL and amplitude controller unstable. On samples with coarse topography and steep features, maintaining stable FM topography feedback demands careful selection of operating parameters and slow scanning speeds. Furthermore, the choice of suitable bandwidths for topography and KFM feedback is more involved in traditional FM-AFM/FM-KFM implementations. For example, when Δ*f* is used as an input to the lock-in amplifier detecting the electrostatic modulation, the PLL bandwidth must be wide enough to include the modulation frequency. Yet, it should be kept as small as possible for stable PLL operation and maximum noise rejection [19]. Finally, the pronounced distance dependence of *C''* for apex and cone, as depicted in Figure 1, makes operation close to the surface more challenging, since small errors of the topography feedback produce marked changes of the effective Kelvin feedback gain. Similarly, when the tip encounters steep edges in topography, *C''* may increase due to a larger effective tip–sample capacitor area, further complicating stable feedback operation. The distance dependence is less pronounced at larger distances employed in lift-mode FM-KFM [10], but in addition to reduced lateral resolution, large modulation voltages are required due to weaker signals [20], which may induce band bending. Furthermore, when scanning across insulating parts of devices, such as gate oxides, not only the local dielectric constant changes, but because of their thickness also a limit is put on the minimum approachable distance in Figure 1. As a result, deliberately slow feedback settings to ensure stable operation are common practice.

In this paper, we describe a practical approach to FM-KFM providing solutions to these issues. We remove the interdependence of topography and KFM feedbacks by focusing on the information contained in the sidebands produced by the electrostatic modulation [20]. Employing a commercially available lock-in amplifier, we detect these sidebands directly. Thus, with frequency modulated distance feedback, the PLL bandwidth can be restricted to the topography only. We further demonstrate the advantage of combining FM-KFM with amplitude modulated AFM (AM-AFM) for tracking topography of highly structured surfaces with small amplitudes and net-attractive interaction in air. Since the oscillation amplitude decreases monotonically with distance, no special precautions are required to ensure feedback stability. Finally, we introduce an improved Kelvin feedback loop based on stochastic optimal control that continuously adjusts its sensitivity to local changes in *C''*, thereby reducing the risk of feedback instabilities and topography crosstalk on difficult heterogeneous samples.

## Theory

### The origin of sidebands

The cantilever motion and the origin of sidebands are understood from a damped harmonic oscillator driven by an external drive, *a*(*t*), and perturbed by the tip–sample interaction force 
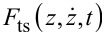
,

[1]
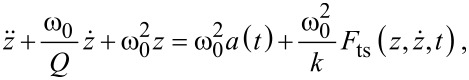


where *z*(*t*) is the cantilever deflection, ω_0_ the eigenfrequency, *k* the spring constant, and *Q* the quality factor of the cantilever. For an oscillation with amplitude *A* and drive frequency ω_d_ ≈ ω_0_, the interaction force can be approximated to


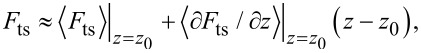


where *z*_0_ is the mean tip position, and 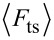 and 
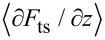
 are the effective force and force gradient, respectively. Explicit expressions for the effective force and force gradient, averaged over the oscillation period, *T* ≈ 2π/ω_d_, are [21]

[2]
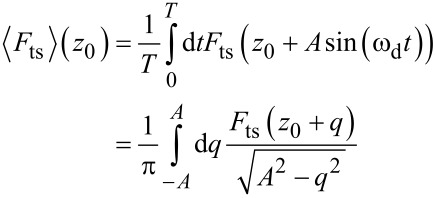


and

[3]
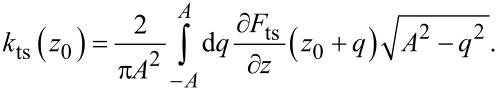


The effective force and force gradient, as introduced here, allow one to describe the motion of the tip in the non-linear force field close to the sample with the model of a perturbed harmonic oscillator, provided the oscillation remains approximately harmonic with constant amplitude [22].

With a small perturbation, *k*_ts_ *<< k*, the resonant frequency of the cantilever changes from ω_0_ to ω_0_ + Δω with Δω/ω_0_ = −*k*_ts_/2*k* [15]. Accordingly, a modulation of the force gradient, e.g., by an oscillating electric field, will cause a frequency modulation of the resonance. A modulation at a single frequency ω_m_ will produce sidebands at integer multiples of the modulation frequency, that is, cantilever oscillations at ω_0_ ± ω_m_, ω_0_ ± 2ω_m_ and so on.

For the derivation of the sideband signals and their respective amplitudes, we assume a modulation of the force gradient at the frequency ω_m_: 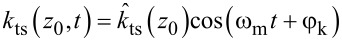. Note that the effective force gradient as calculated above, Equation 3, is valid for ω_m_ *<<* ω_d_.

Then, by Fourier transformation of the equation of motion, Equation 1, we arrive at

[4]
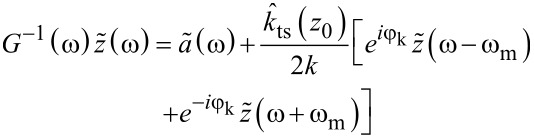


with

[5]
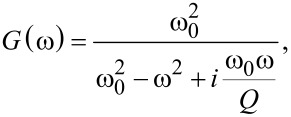


where 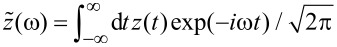 and 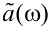 are the Fourier transformed deflection and drive, respectively, and *G*(ω) is the complex transfer function of the damped harmonic oscillator.

Equation 4 and Equation 5 present an iterative scheme to determine the spectral components of the cantilever oscillation, where in each step 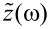 on the left hand side of Equation 4 is refined by the expressions on the right hand side. Starting from an oscillator at rest, 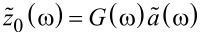 is the carrier oscillation due to the external drive, as in the unperturbed system. Spectral components at ω ± ω_m_ emerge in the next iteration step,

[6]
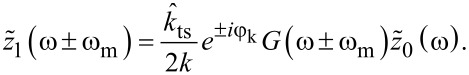


This is the fundamental pair of sidebands of the force modulated damped harmonic oscillator. With 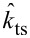
*<< k*, the higher order sidebands arising in the subsequent iterations are usually negligible.

Equation 6 also describes the sideband amplitude transfer function when the expression is evaluated close to the sideband frequencies. With the substitution ω − ω_d_ → ω, we find

[7]
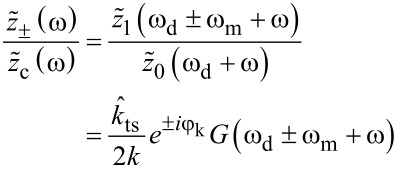


with the approximation

[8]
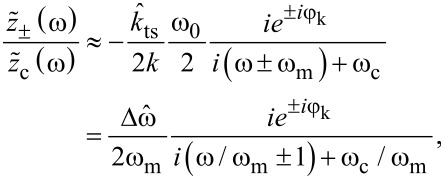


where, in the latter approximation, we consider only the dominant term for a drive close to the eigenfrequency, i.e., ω_d_ ≈ ω_0_ and ω *<<* ω_0_, and ω_c_ = ω_0_/2*Q* is the cantilever bandwidth.

For modulation frequencies well beyond the cantilever bandwidth, *G*(ω_0_ ± ω_m_) ≈ *−iω*_0_/2ω_m_, and the amplitude of each sideband is 
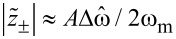
, where *A* is the carrier amplitude. The latter expression also follows immediately from a narrow-band frequency modulation of a carrier oscillation at ω_d_. With a carrier amplitude *A* and the peak frequency deviation 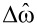, a frequency modulation at ω_m_ produces two sidebands with amplitudes β*A*/2, where 
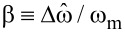
 is the modulation index [23]. Under ultra-high vacuum conditions, large *Q* factors typically cause negligible cantilever bandwidths, making this approximation valid, e.g., for finding the noise power spectral density of the frequency shift signal in FM-AFM [24]. When the narrow-band conditions are not met (β *>>* 1), the iterative scheme for the sideband amplitudes in Equation 4 and Equation 5 still approaches the Bessel functions describing the sideband amplitudes in a general frequency modulation for ω_m_ *>>* ω_c_ (see Supporting Information File 1).

**Figure 2:**
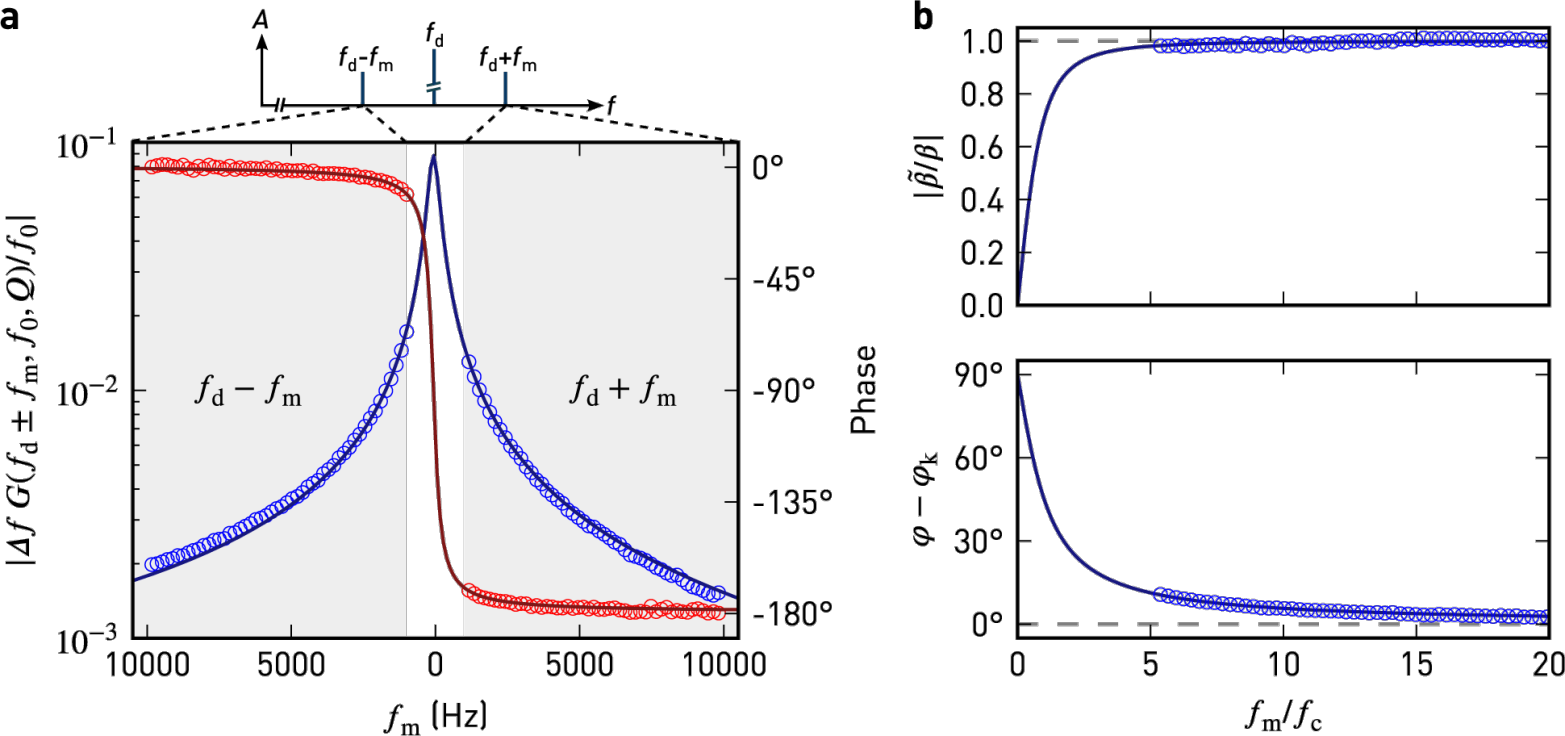
a) Sideband amplitude (blue) and phase (red) relative to the carrier oscillation measured during a sweep of the modulation frequency (markers), and a fit of the harmonic oscillator (solid lines). At a constant height of approx. 25 nm above the sample, the tip was driven mechanically at *f*_d_ = ω_d_/2π = 70657 Hz and electrically at *f*_m_. Parameters to the least-squares fit are 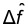 = 33 Hz, *f*_0_ = 70586 Hz, and *Q* = 190. b) Amplitude and phase of the complex modulation index for a narrow-band frequency modulation, 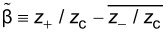. The solid lines are a plot of the approximation in Equation 9. The dashed lines indicate the expected behaviour for *f*_m_*>> f*_c_, neglecting the influence of the damped harmonic oscillator.

The Fourier approach presented above also accurately models the behaviour of the sideband amplitude and phase for modulation frequencies approaching the cantilever bandwidth ω_c_. In Figure 2a, we show the expected and experimentally measured sideband amplitudes and phases. The excellent agreement with the above model proves the validity of our derivation. Each sideband is phase-shifted by ±φ_k_ + arg*G*(ω_d_ ± ω_m_).

For narrow-band frequency modulation, we can define a complex modulation index 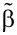 by the sideband and carrier amplitudes as 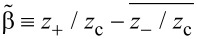. With Equation 8, the dc response (ω → 0) of 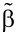 thus is

[9]
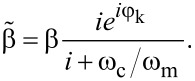


Figure 2b shows the amplitude and phase of 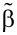 for pure narrow-band FM and the harmonic oscillator as a function of ω_m_/ω_c_. The amplitude and phase only agree with the result for pure narrow-band frequency modulation when the cantilever bandwidth is negligible compared to the modulation frequency. For low modulation frequencies 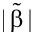 approaches 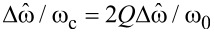 instead (Equation 9).

**Figure 3:**
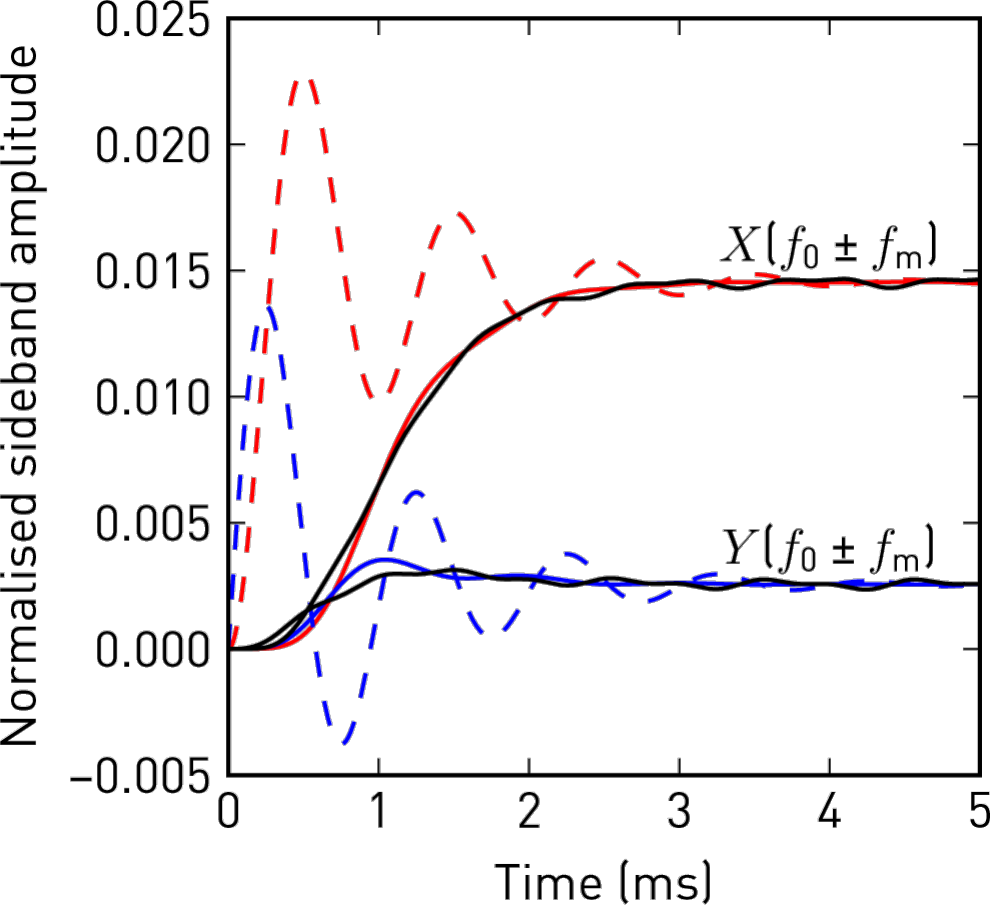
In-phase (red) and quadrature (blue) sideband amplitudes, normalised to the carrier oscillation amplitude *A*, in response to a step in the force gradient modulation amplitude. The step responses following from Equation 8 (dashed), show oscillations at *f*_m_, exponentially decaying with 1/ω_c_, which are removed by the low-pass filter of the lock-in amplifier (solid, *f*_cut_ = 250 Hz, 24 dB/oct). The solid black lines show the demodulated sideband amplitudes from a direct numerical simulation of the perturbed harmonic oscillator. (Numerical parameters: *f*_0_ = 70500 Hz, *Q* = 200, *A* = 10 nm, *f*_m_ = 1000 Hz, φ_k_ = 0, 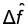 = 30 Hz).

To further demonstrate the validity of the sideband transfer function, we show in Figure 3 the response to a step in 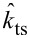 from both the approximation in Equation 8 and from a numerical simulation of the perturbed harmonic oscillator, Equation 1, including lock-in amplifiers at ω_d_ ± ω_m_. Each change in the force gradient modulation also excites a transient oscillation at the resonant frequency of the cantilever, which appears in the sideband signal and decays exponentially with 1/ω_c_. Therefore, the filter settings of the lock-in amplifier should be set accordingly to provide sufficient rejection near ω_m_.

We conclude that sidebands evolve as soon as *k*_ts_ gets modulated and it is not important whether the resonant frequency is actually tracked or not. The main benefit of tracking the resonant frequency (e.g., with a phase-locked loop) is merely to keep the carrier phase constant, which would otherwise affect the sideband phases.

### Electrostatic force and force gradient

The electrostatic force between the AFM tip and sample is


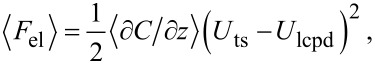


where 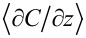 is the effective capacitance gradient, *U*_ts_ is the tip–sample voltage, and *U*_lcpd_ is the local contact potential difference.

For Kelvin probe force microscopy, *U*_ts_ is modulated around a dc voltage: *U*_ts_ = *U*_dc_ + *U*_ac_ cos(ω_m_*t*). Therefore, the electrostatic force and likewise its gradient, 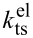, are modulated at ω_m_ and 2ω_m_,


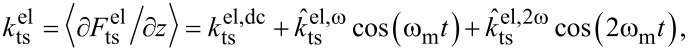


where

[10]
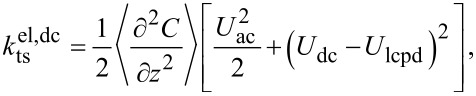


[11]
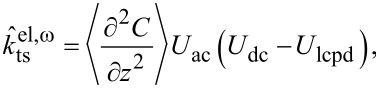


and

[12]
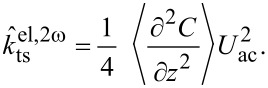


These modulations of the force gradient cause sidebands of the cantilever deflection at ω_d_ ± ω_m_ and ω_d_ ± 2ω_m_, which can be detected directly with lock-in amplifiers at the respective frequencies. The lock-in amplifiers return, relative to the reference oscillator, amplitude and phase of each sideband as well as their cartesian projection: the in-phase component *X* and the quadrature component *Y*. In the narrow-band approximation for ω_m_ *>>* ω_c_, the in-phase components of the modulation at ω_m_ and the amplitudes at 2ω_m_ are

[13]
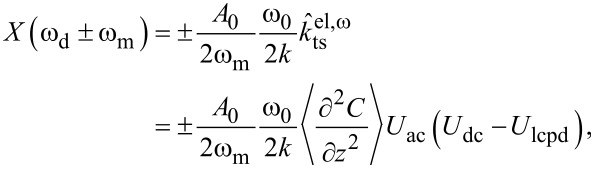


and

[14]
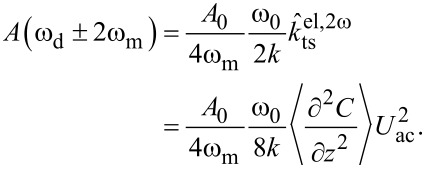


The reference phase offsets of the lock-in amplifier for the first set of sidebands at ±ω_m_ are chosen to maximise their respective in-phase components, taking into account the 180° phase shift of the lower sideband. Then, *X*_ω_ = *X*(ω_d_ + ω_m_) − *X*(ω_d_ − ω_m_) = 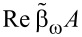 is the total in-phase component, which depends linearly on the applied dc bias. Furthermore, when *U*_dc_ matches *U*_lcpd_, *X*_ω_ is nullified and the ±ω_m_ sidebands disappear.

The total amplitude of the second set of sidebands, *A*_2ω_ = *A*(ω_d_ + 2ω_m_) + *A*(ω_d_ − 2ω_m_) = 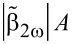, only depends on the ac modulation amplitude and the second order capacitance gradient, 
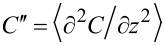
. This signal thus provides a handle for imaging variations in the tip–surface capacitance, surface dielectric properties [25], or lateral dopant profiling [26].

**Figure 4:**
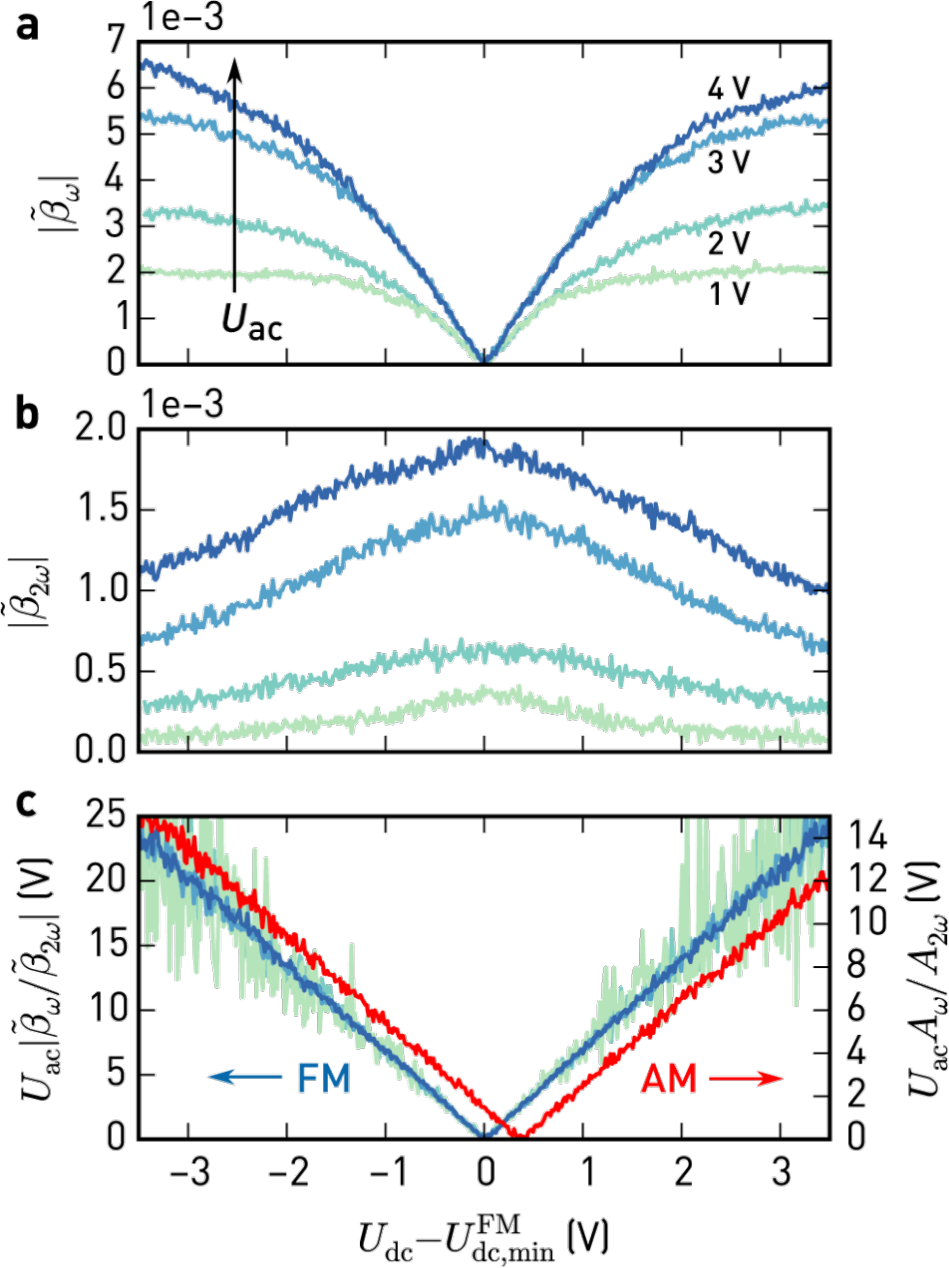
Modulation indices of the sidebands at ω_m_ (a) and 2ω_m_ (b) against 
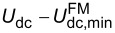
 for different modulation amplitudes *U*_ac_. Topography feedback in amplitude modulation (net-attractive interaction) was enabled during these measurements. 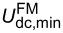, given by the minimum of 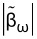, is the contact potential difference found by FM detection. c) Signals in (a) normalised using *U*_ac_ and 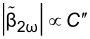 (light green to blue, FM). Normalised electrostatic force (red, AM), simultaneously detected with lock-in amplifiers at ω_m_ and 2ω_m_. (Scan parameters: *A*_free_ = 11.2 nm, *A*_set_ = 10.4 nm, *Q* = 500, *k* = 35 N/m, *f*_0_ = 302.5 kHz, *f*_m_ = 4 kHz).

In Figure 4, we show experimental data of modulation indices 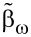 and 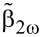, calculated from the ω_m_ and 2ω_m_ sidebands, respectively, as a function of *U*_dc_ for different electrostatic modulation amplitudes, *U*_ac_. During this experiment, the tip was positioned above a nickel electrode with amplitude modulated topography feedback enabled in net-attractive mode.

As expected from Equation 13, the ω_m_ sideband amplitudes (Figure 4a) vanish when *U*_dc_ = *U*_lcpd_. While they change linearly with *U*_dc_ close to this point, there are non-linear deviations at larger voltage offsets, which are caused by changes in *C''*. This is also evident from the 2ω_m_ sideband amplitudes (Figure 4b), showing the decrease of *C''* with increasing voltage offsets. Since these sweeps are acquired with topography feedback enabled, the observed variations in *C''* are most likely due to changes in the tip–surface separation: The AM topography feedback is sensitive to the static force gradient, which contains electrostatic interactions, Equation 10, that increase as the dc bias does not match the surface potential; consequently, the topography feedback retracts the tip, reducing *C''*.

In Figure 4c, we plot the ratio of the ω_m_ and 2ω_m_ sideband amplitudes, normalised to *U*_ac_. As apparent from Equation 13 and Equation 14, this process cancels out the non-linearities and collapses the sweeps at different *U*_ac_ to a single curve.

Additionally, we show the similarly normalised amplitudes due to the electrostatic force at ω_m_ and 2ω_m_ in the deflection signal, which we acquired simultaneously with the sidebands at ω_d_ ± ω_m_. They show the same v-shaped relationship, with their minimum being slightly shifted with respect to the FM case. This shift is due to the different weights of contributions in the AM signal (cf. Figure 1). Setting the dc bias to the minimum obtained by AM-KFM does not guarantee to compensate the electrostatic force gradient and can cause height errors in topography. At the minimum determined from the sidebands, 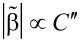 in Figure 4b reaches its maximum value, corresponding to the closest approach.

There are two major methods to find the local contact potential difference at every point during the scan. *Open-loop* KFM exploits the fact that the 2ω_m_ amplitudes do depend on *C''* but not on *U*_dc_ − *U*_lcpd_. As demonstrated in Figure 4, the ratio of the ω_m_ and 2ω_m_ sidebands is independent of changes in *C''* and only depends on the chosen modulation amplitude and dc bias,

[15]
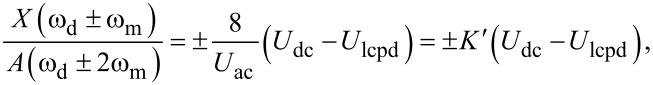


hence *U*_lcpd_ can easily be determined. Note, however, that the above definition of the prefactor *K*^′^ is only valid for modulation frequencies well beyond the cantilever bandwidth. In the general case, *K*^′^ = (4/*U*_ac_) *G*(ω_d_ ± ω_m_)/*G*(ω_d_ ± 2ω_m_), that is, it also depends on the resonant frequency and the quality factor, which may change while scanning. Furthermore, there may be differences in the sideband phase shift when *Q* or ω_0_ are not constant (cf. Figure 2). Together, such inaccuracies in the model easily translate into uncertainties of *U*_lcpd_ in an open-loop method. A PLL can reduce these effects, but then its transfer function needs to be considered as well [27], and the bandwidth must be larger than 2ω_m_.

In *closed-loop* KFM, the local contact potential difference is found by nullifying the in-phase components of the ω_m_ sidebands (Equation 13) with a feedback loop adjusting the applied dc voltage [1–3,16]. Thus, the 2ω_m_ sidebands are not necessary to determine the CPD, and the effect of model deviations and non-linearities is cancelled by the feedback. Furthermore, the nulling process also minimises the dc electrostatic force and force gradient (Equation 10), reducing electrostatically induced height errors [14,28–29].

However, a few critical issues remain with simple Kelvin feedback loops. For example, when the sidebands are not completely nullified by the feedback, leaving a small error δ, it follows from Equation 13 that 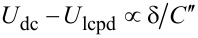
[30]. *C''* depends strongly on the electrostatic interactions between tip and surface and may change significantly on structured surfaces even for a well-tuned topography controller. During a scan, imperfect Kelvin feedback therefore leads to errors in the measured CPD, constituting a source of topography-induced crosstalk.

If additional apparent forces (or force gradients) are detected at the frequencies used for KFM, the Kelvin feedback does not compensate the CPD, but rather nullifies the in-phase component affected by offsets [31]. Such crosstalk is due to parasitic capacitive coupling and observed mainly in AM-KFM, where the electrostatic modulation is at high frequencies [32]. When coupling to the shaker piezo [33], cantilever resonances can amplify this effect.

Another source of crosstalk can appear when ω_m_ is set too low and the Kelvin lock-ins capture the modulation of *k*_ts_ induced by topography. This can happen on highly structured surfaces when the bandwidth of the topography feedback is insufficient for the scan speed. By monitoring the deflection power spectral density near the driving frequency, an upper frequency bound of the remaining *k*_ts_ modulations can be determined. In order to avoid crosstalk, ω_m_ should be chosen above this bound, considering both the bandwidth and filter steepness of the Kelvin lock-ins.

As already mentioned above, the tuning of the Kelvin feedback loop itself can be a challenge because its sensitivity depends on *C''*. This becomes even more acute for small tip–sample distances and single-scan techniques on structured surfaces, where the tip–surface interaction is not limited to the apex.

In order to address the topography crosstalk due to *C''*, Lee et al. [34] suggested to use a feedback signal normalised to the 2ω_m_ sideband, thus rendering the CPD tracking error independent of *C''* (cf. Equation 15). However, as shown in Figure 4c, the normalisation procedure may introduce additional noise when dividing by small signals, e.g., for low *U*_ac_.

In the following section we introduce a novel Kelvin feedback scheme that resolves these subtleties.

## Results and Discussion

### Optimal CPD estimation and Kelvin control

**Figure 5:**
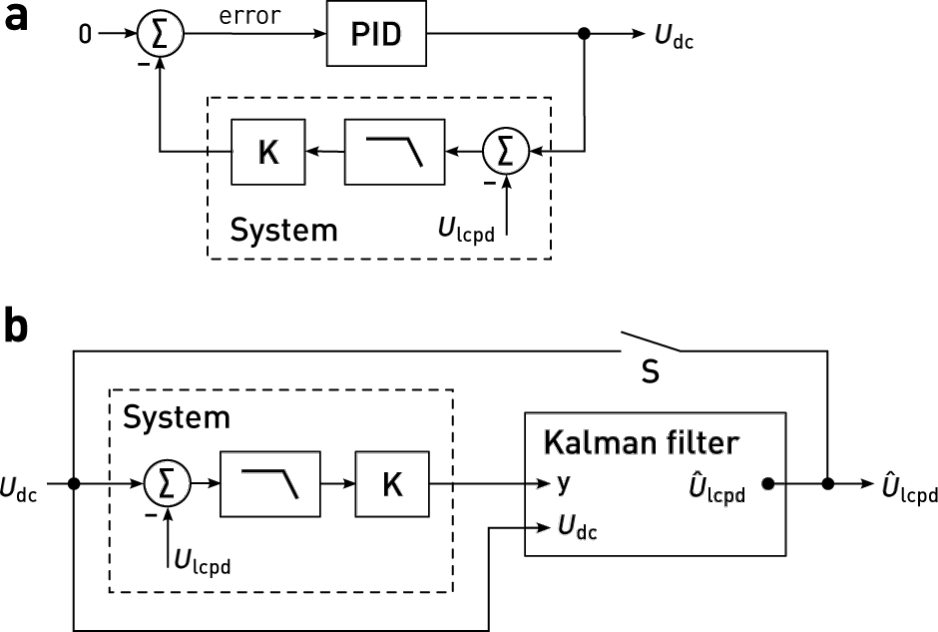
Block diagram of a Kelvin controller based on a) a proportional–integral–differential (PID) controller, and b) the Kalman filter. In the PID controller, the system output is compared to a setpoint to yield the error signal. The output signal, i.e., the sum of amplified errors and their respective integral or derivative, is fed back into the system. When the system output is nullified (setpoint 0), the controller output *U*_dc_ equals the surface potential *U*_lcpd_. In contrast, knowing an approximate model of the system, the Kalman filter estimates 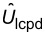 solely based on the system output and the applied dc bias, *U*_dc_. With the switch *S* closed, the estimated surface potential 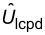 is applied as the dc bias, corresponding to a feedback configuration.

Most instruments provide a generic PID controller for Kelvin control, which compares the signal (*X*_ω_) to a setpoint (0), yielding the error signal *e*. The sum of *e*, ∫d*te*, and d*e*/d*t*, scaled by respective proportional (P), integral (I), and derivative (D) gains, is fed back into the system. In case of KFM, the resulting dc voltage compensates the electrostatic interactions. This standard PID feedback loop is illustrated in Figure 5a. Knowing the system dynamics, a multitude of tuning rules can be applied [35]. In practice, however, the feedback gains are often tuned by trial and error, and the derivative part is omitted altogether [36]. In many cases, only the integral part is necessary for good tracking and to eliminate steady-state errors. Integral-only controllers are therefore prevalent for topography or Kelvin feedback.

Controllers basing their actions on an error signal only are unaware of the systems they control. Thus, they need to be retuned as soon as the system bandwidths or gains change considerably, either due to different operator settings or, more importantly during KFM scans, due to local variations of electronic properties and topography of the sample. To maintain best feedback settings at every location during a scan, we introduce a novel controller for FM-KFM based on stochastic optimal control [37]. Optimal control and model-based controllers have been successfully used before in AFM, e.g., for active damping of cantilevers [38] or fast scanning [39]. According to the separation principle [37], the optimal controller that minimises the expected error can be constructed by finding an optimal ‘observer’ and an optimal ‘regulator’. As an observer, we use a Kalman filter [40], which continuously blends the sideband measurements at ±ω_m_ into an estimate of the contact potential difference, 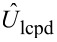, based on a simplified model of the FM-KFM detection system. The Kalman filter is the stochastically optimal observer that minimises the state error covariance [37], taking into account both measurement noise and the uncertainties in the knowledge of its state. Adapted for KFM control, the Kalman filter minimises the estimation error variance of the surface potential, 
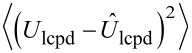
. Since the aim of the regulator in KFM is to minimise electrostatic interactions, the optimal regulator is found by matching the dc bias to 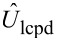, thereby closing the feedback loop (Figure 5b).

Our Kalman filter design based on a model of the KFM detection system includes the sideband dynamics, Equation 8, the electrostatic force gradients acting on the cantilever, Equation 11 and Equation 12, and the transfer function of the demodulating lock-in amplifier. Since the lock-in bandwidths must be kept well below ω_m_ to avoid carrier and topography crosstalk, the sideband transfer functions reduce to an effective gain and phase, Equation 9. The lock-in transfer function can either be measured or is known from its filter properties. We focus on a particularly simple case, the *n*-th order critically damped lowpass filter, which is formed by *n* consecutive first order stages with a time constant τ. With these considerations, the transfer function for the in-phase lock-in components is *G*(*s* = *iω*) = *K*(1 + τ *s*)*^−n^*, where, following Equation 13, we find the static gain 
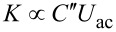
 and the system output *G*(*s*)(*U*_dc_ − *U*_lcpd_).

Based on the transfer function, we find a state-space model of the system, in which we incorporate *U*_lcpd_ as a *hidden* state, and *U*_dc_ is the control signal. We further model the uncertainties of state transitions (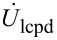) and our measurements as uncorrelated, zero-mean white noise with power spectral densities *V* and *W*, respectively. Hence, *U*_lcpd_ follows a Wiener process or Brownian motion [37]. For a derivation of the continuous-time Kalman(-Bucy) filter [41], see Supporting Information File 1. In discrete time, the Kalman filter is similarly found from a discrete-time state-space model [40]. In this formulation the state estimate and covariances are refined recursively as new measurements are incorporated:

At the time *t*, an a priori state and covariance estimate is found using the state and covariances at the time *t* − Δ*t*, based on the system model. Then, the Kalman gain *L* is computed from the covariance matrices of the a priori estimated state and the system model. *L* controls the innovations process, in which the measurements at the time *t* are incorporated to the a posteriori estimate of state and covariances [42].

**Figure 6:**
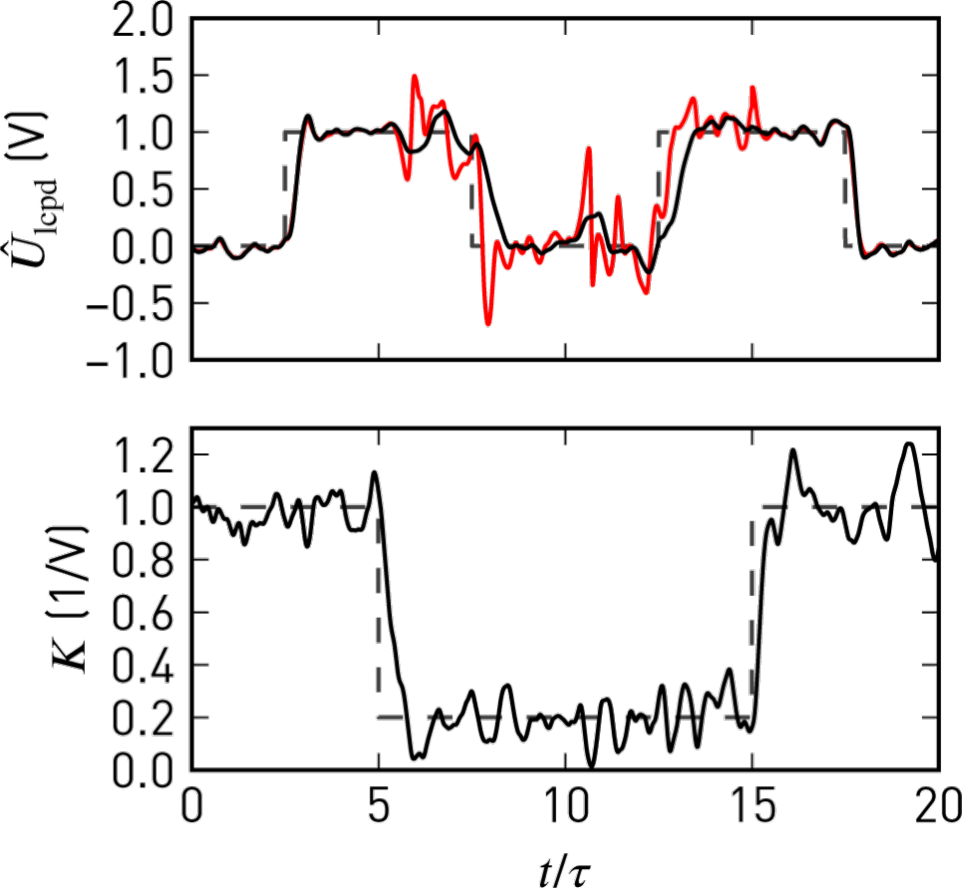
Closed-loop response of the Kelvin observer (black) and a proportional-integral controller (red) to steps in *U*_lcpd_ and *K*. Both controllers incorporate the separately measured static gain, *K*, and are tuned for similar step responses at high *K*. Noise in *K* and at the inputs is artificial white noise lowpass-filtered with τ*f*_cut_ = 1 and the filter order *n* = 4, corresponding to the simulated system.

This recursive predictor–corrector structure allows for updates of the system parameters, such as the static gain *K*, at each instant of the state update. With Equation 15, the 2ω sideband amplitudes can thus be exploited to continuously update *K* = *K'A*_2ω_. Consequently, the observer model will follow changes in the Kelvin signal strength due to variations of *C''*. This strategy avoids normalisation by potentially noisy *C''* signals [34], yet changes in *C''* do not affect closed-loop performance. We demonstrate this in Figure 6, where we compare step responses of the closed-loop Kalman observer and PI controller. As soon as the gain *K* drops, the noise level increases with a PI controller, whereas the Kalman estimate remains clean.

**Figure 7:**
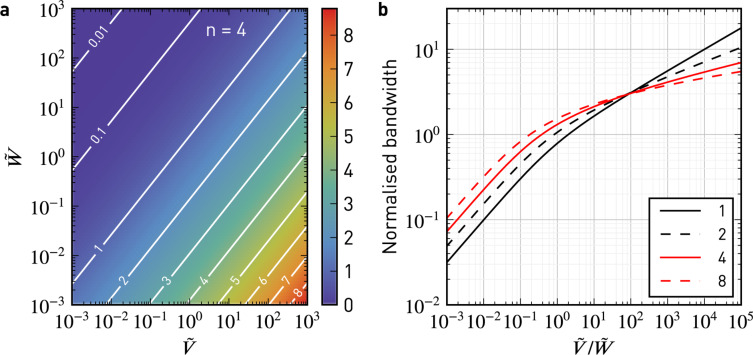
Normalised closed-loop bandwidth (−3 dB) of the steady-state Kelvin observer as a function of the normalised power spectral densities 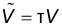 and 
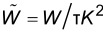
 for different orders of the low-pass filter, *n*. a) Two-dimensional map for *n* = 4. As the observation noise density, 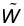, increases for a fixed transition noise density, 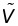, the closed-loop bandwidth is reduced. b) Closed-loop bandwidth normalised to the bandwidth of the corresponding *n*-th order low-pass filter.

To further elucidate the performance of the controller, we plot in Figure 7 its −3 dB closed-loop bandwidth, normalised to the −3 dB filter bandwidth, as a function of the normalised noise power spectral densities 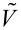 and 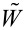 of state transitions and observations, respectively. As the noise at the output, 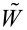, increases for a fixed 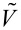, the bandwidth is reduced (Figure 7a). The ratio 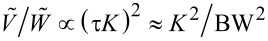 resembles a signal-to-noise ratio (SNR), which increases for large *K* and small filter bandwidths BW. The closed-loop bandwidth is a function of this SNR. Therefore, in addition to avoiding divisions by small signals, the Kalman filter improves noise performance by bandwidth adjustments. For normalised closed-loop bandwidths ≤ 1, the bandwidth is adjusted following 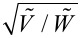 (Figure 7b). Larger bandwidths are not desired, since they would counteract the lock-in lowpass action.

**Figure 8:**
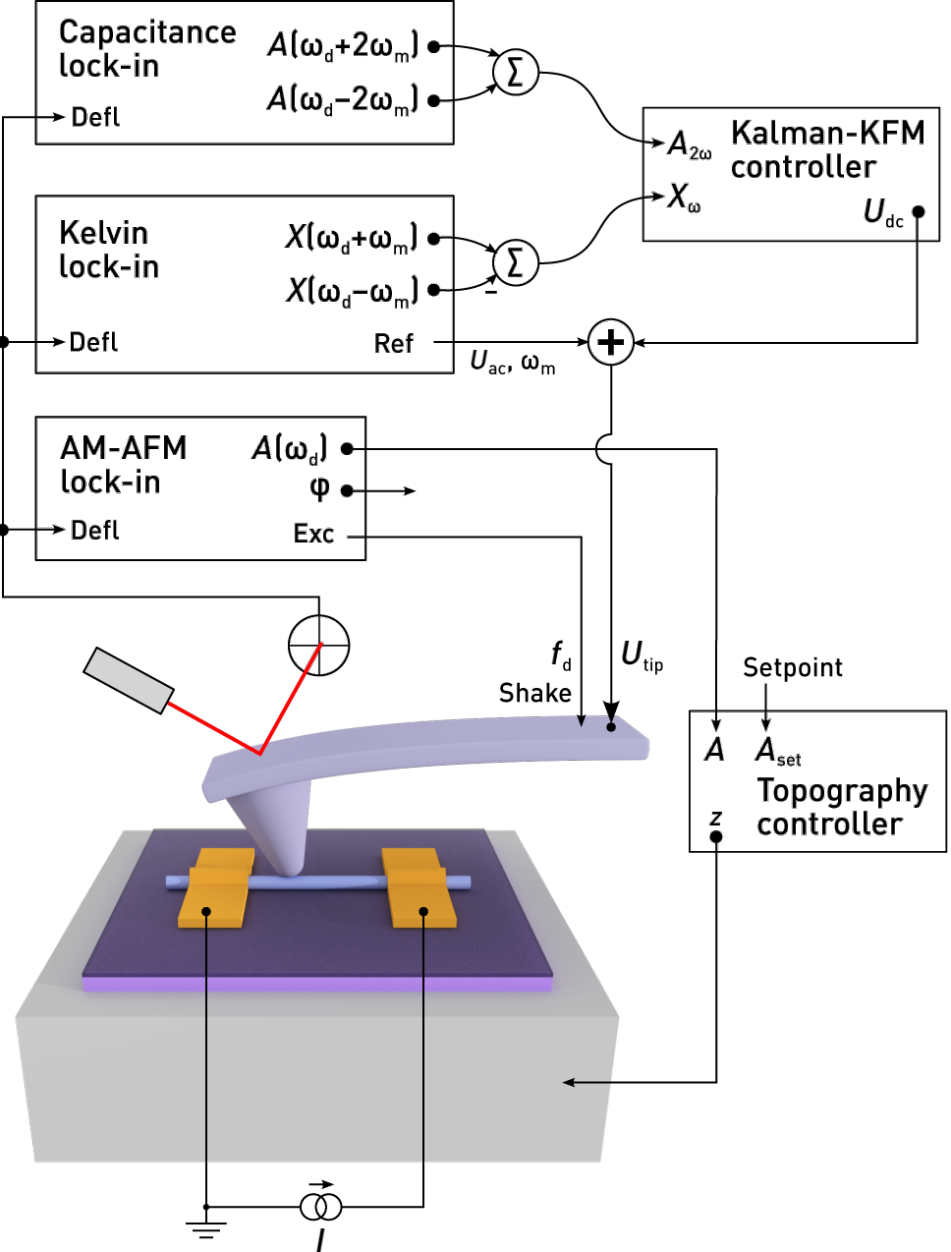
Schematic of the modified KFM setup. For topography feedback, the cantilever is excited at a constant frequency ω_d_ close to resonance. Lock-in amplifiers at ω_d_ ± ω_m_ and ω_d_ ± 2ω_m_ detect sidebands of the cantilever oscillation which contain information about the surface potential and tip–sample capacitance. Both contributions are used by the Kalman-KFM controller for the CPD estimate.

Our setup is shown in Figure 8. We implemented the Kalman-filtering Kelvin controller as a real-time program on the digital signal processor (DSP) of a digital lock-in amplifier and PLL (HF2, Zurich Instruments), which demodulates the sidebands at ω_d_ ± ω_m_ and ω_d_ ± 2ω_m_ as well as the carrier signal at ω_d_. Since our implementation of the Kalman filter is integrated into the lock-in, all signals are available without additional digital/analog/digital conversions. Additional offsets that might affect the feedback accuracy are avoided. We have implemented the Kalman filter as a reusable component in C++ using the Eigen template library for linear algebra [43], allowing us to perform offline tests with the same code that is compiled for the DSP. In its current state, our custom FM-KFM controller can work at sampling rates of up to 7200 Sa/s.

Since the sideband signals are detected individually, we do not depend on the Δ*f* signal as in a typical FM-KFM setup. Therefore, the Kelvin feedback remains the same for AM and FM topography feedback schemes. For example, on samples with coarse topography one may use AM topography feedback to avoid instabilities typically experienced with FM operation. In vacuum, this may require additional application of active *Q* control [44–45] to lower the *Q*-factor of the cantilever.

### Performance on a nanowire device

**Figure 9:**
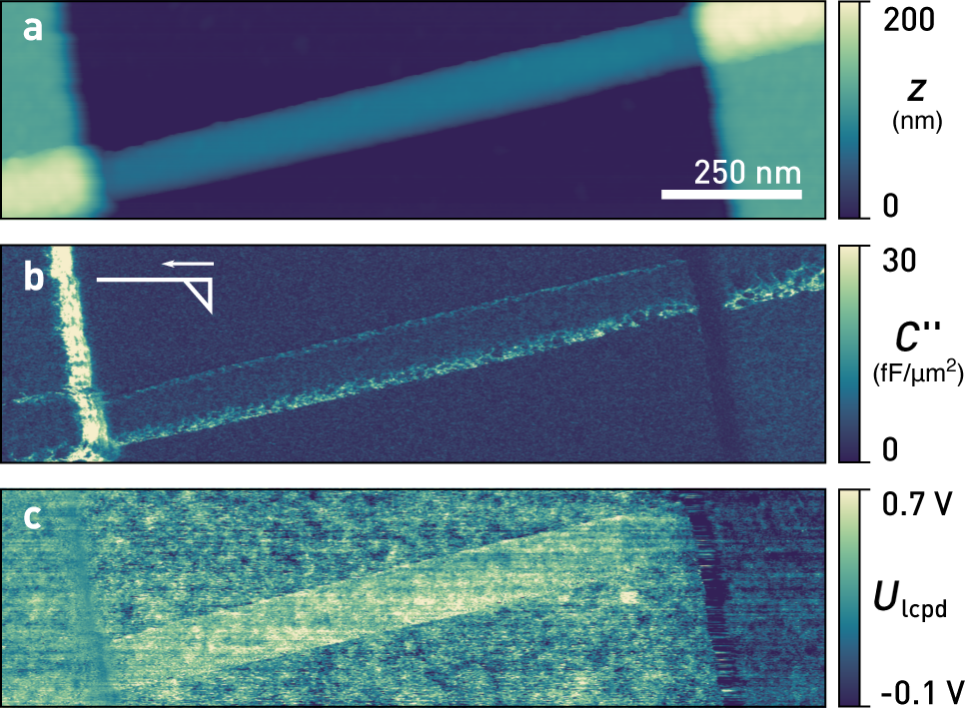
a) Topography, b) tip–sample capacitance gradient, *C''*, and c) local contact potential difference, *U*_lcpd_, of an InAs nanowire at a bias current of 1.4 μA. *U*_lcpd_, determined by the Kelvin observer, exhibits no crosstalk. The inset in b) indicates the tip shape and fast scan direction. (Scan parameters: *A*_free_ = 8.6 nm, *A*_set_ = 7.2 nm, *Q* = 390, *k* = 26 N/m, *f*_0_ = 304.2 kHz, *f*_m_ = 4 kHz, *U*_ac_ = 1 V, *f*_cut_ = 100 Hz, *n* = 4, *v*_tip_ = 800 nm/s).

To demonstrate the performance of our Kalman-KFM controller, we examine an active nanowire device as depicted in Figure 8 and Figure 9. Such devices exhibit some of the most typical and relevant issues hindering reliable KFM measurements in the past: a combination of large topography with a multitude of different materials including oxides prone to charging. In Figure 9, we show a scan of a 70 nm diameter indium arsenide (InAs) nanowire with nickel (Ni) contacts (height ≈ 120 nm), obtained at a bias current of 1.4 μA under ambient conditions using a commercial AFM (Cypher, Asylum Research). The steep edges at the electrodes necessitated AM topography feedback. Oftentimes the contact resistances between nanowire and metal contacts are uneven and large, obscuring the electrostatics of devices. Traditional four-point measurements are limited at sub-micron length scales because the contact length can become comparable to the channel dimensions. For such samples, KFM appears to be an ideal tool to characterise the electrostatics in order to optimise device performance, for example as field effect transistors.

Figure 9b displays the simultaneously acquired *C''*, calculated from the 2ω_m_ sideband amplitudes, Equation 14. To ensure highest lateral potential resolution, we used highly doped silicon AFM tips (Olympus AC160TS-R3) without a metal coating. These tips are sharp and not symmetrical at the apex (schematically depicted in the inset), explaining the increased *C''* on the edge of the left electrode.

Figure 9c shows 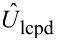 as estimated by the Kalman-KFM controller. Since its gain is continuously updated using the 2ω_m_ sidebands, crosstalk due to changes of *C''* is absent from the scan. Near the left electrode edge the measured *U*_lcpd_ displays less spatial variation because also the sides of the tip are in close proximity to the electrode edge, increasing their contribution to the tip–sample capacitance and widening the KFM point spread function. Even though the Kalman-KFM controller remains stable and works unaffected by the increased *C''*, reaching up to seven times the mean value of the scan, the geometry of both tip and sample fundamentally limit the attainable resolution. The disturbances remaining on the edge of the right contact are due to imperfect topography feedback and accidental switches from net-attractive to net-repulsive tip–sample interactions. Most importantly, edge effects are absent at the boundaries of the nanowire. Long-range potential averaging due to the cantilever beam is absent due to the gradient-sensitive FM detection.

For an extraction of contact resistances, the voltage profile due to current flow needs to be separated from additional offsets in *U*_lcpd_, such as spatial variations in work function. These are easily obtained from a scan at zero bias. Knowing the potential drop at the contacts, the bias current, and assuming uniform material or transport properties, for the nanowire device in Figure 9 we hereby obtain a contact resistance of 40 kΩ at the left electrode, 150 kΩ at the right electrode, and a channel resistance of 50 kΩ.

**Figure 10:**
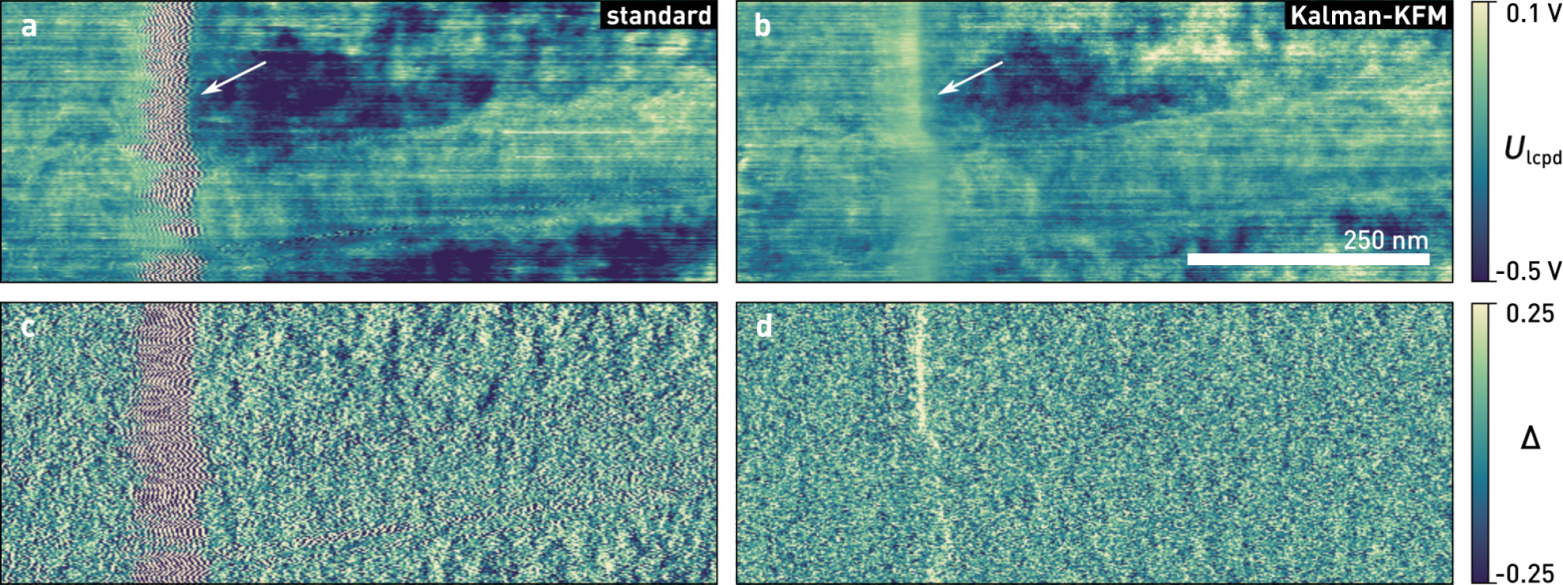
a), b) Kelvin and c), d) error signal of an InAs nanowire similar to the device shown in Figure 9, measured with a standard integral controller, a) & c), and the Kelvin observer, b) & d). Feedback oscillations, as indicated by the arrow, are absent in the Kalman control scheme, while the standard deviation of the error signal also decreases from 0.19 to 0.13. (Scan parameters: *A*_free_ = 11.2 nm, *A*_set_ = 9.6 nm, *Q* = 500, *k* = 35 N/m, *f*_0_ = 304.5 kHz, *f*_m_ = 4 kHz, *U*_ac_ = 2 V, *f*_cut_ = 50 Hz, *n* = 4, *v*_tip_ = 250 nm/s; all images show raw data).

In Figure 10, we compare the performance of our Kalman-KFM controller with a standard integral controller. Both controllers are tuned for optimised closed-loop performance on the nanowire. The integral controller exhibits ringing artefacts at electrode edge, indicated by an arrow in Figure 10a, since the gain margin of the controller is exceeded due to the increased *C''*. Such feedback oscillations should be avoided particularly in single-scan techniques, because they may perturb the topography controller. Next to the nanowire, where *C''* is slightly reduced because of the gate oxide, the bandwidth of the feedback loop drops due to a lower gain.

**Figure 11:**
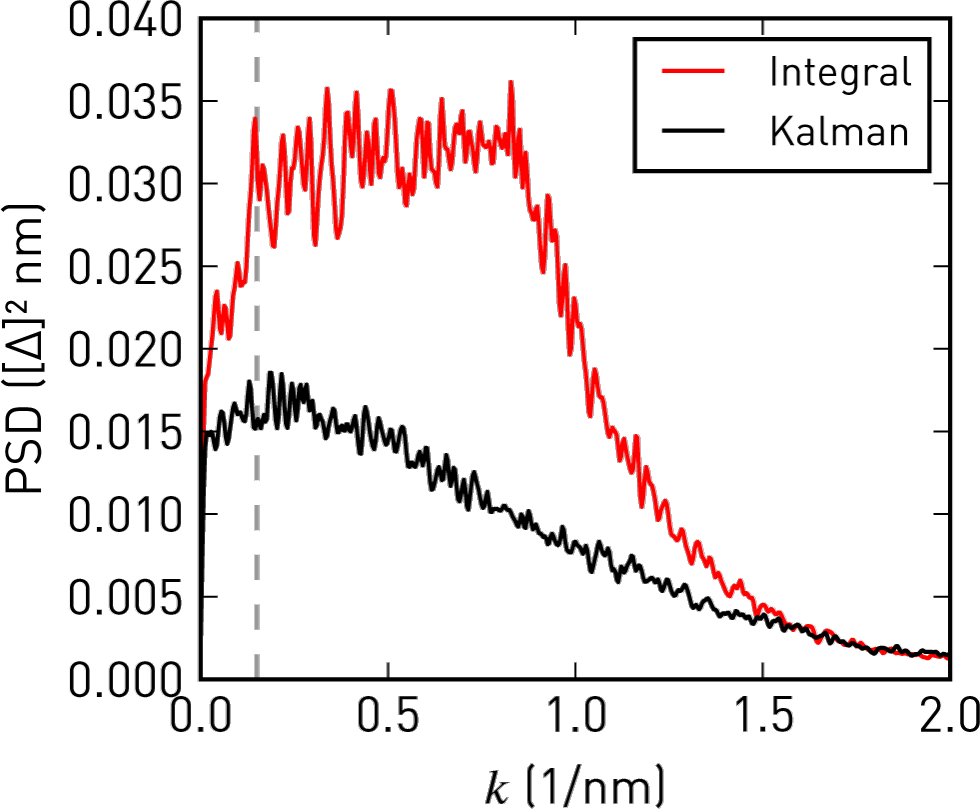
One-dimensional power spectral densities of the error signals in Figure 10c,d. Integral feedback works well at low spatial frequencies, *k*, but is unable to follow higher-frequency modulations. Kalman-KFM control consistently shows a lower error signal at all frequencies. In both cases the roll-off at high *k* is due to the Kelvin lock-in bandwidths.

As shown in Figure 10b, with the Kalman-KFM controller the feedback performance and image quality remain consistent during the scan. The error signal (*X*_ω_) is almost feature-less and its standard deviation reduces by about 30% on average (excluding edge effects). Better tracking is also apparent from the power spectral densities of the error signals, depicted in Figure 11. For given lock-in filter bandwidths, the Kalman-KFM controller can nullify the ±ω_m_ sidebands faster and better than the integral controller, without adding to the noise level or introducing feedback artefacts.

## Conclusion

We have demonstrated a novel feedback controller for high resolution, frequency modulated Kelvin probe force microscopy. Based on Kalman filtering and stochastic optimal control, our feedback employs a model-driven estimation process, which allows one to integrate sensitivity information from the 2ω_m_ sidebands. In comparison to normalisation approaches [34], this strategy does not increase the noise level.

We have tested performance on an InAs nanowire device with rough surface and abrupt height variations, which pose severe challenges to both traditional single-scan and lift-mode FM-KFM setups. Since direct sideband demodulation allowed us to perform FM-KFM irrespective of the topography feedback, we could perform these scans with amplitude modulation in air. Similarly, Magonov and Alexander [46] demonstrated a setup in which the modulated force gradients are detected from the phase output of the carrier oscillation lock-in, requiring ω_m_ to be within its bandwidth. With direct sideband detection, the detour via a phase modulation is avoided, and ω_m_ can be chosen independently of the lock-in bandwidth to achieve best separation from topography.

We have provided a detailed quantitative description of the evolution of sidebands in dynamic AFM modes. Precise knowledge of their frequency dependence in low and high *Q* environments is not only neccessary for accurate open-loop KFM techniques, but also offers a direct approach to noise performance and optimisation of frequency modulated KFM [19]. For example, ω_m_ should ideally be chosen below the thermal noise limited bandwidth of the cantilever [24], but the modulation induced by rough surfaces as well as the desired scan bandwidth establish lower limits. Furthermore, the sideband transfer function explains the higher resolution obtained by heterodyne amplitude-modulated KFM [47]. In this technique, the cantilever is driven mechanically at ω_0_ and electrically at ω_m_ = ω_1_ − ω_0_, where ω_0_ and ω_1_ are the lowest two eigenfrequencies of the cantilever. Accordingly, the sideband at ω_0_ + ω_m_ coincides with the second eigenmode of the cantilever, resulting in an amplified signal proportional to the electrostatic force gradient instead of the electrostatic force.

Although we have found our feedback loop superior to existing controllers, we see several aspects for improvement in the future. For example, the dynamics of *U*_lcpd_ are currently modelled as white noise. Since successive lines in AFM scans only change slightly, information from the previous line could be incorporated, similar to a feed-forward controller [29]. Other state estimators could also be integrated, including H-∞ filters for minimising the worst-case error [42].

Finally, we would like to point out that using our Kalman-KFM controller is not complicated. Since it automatically incorporates the lock-in filter settings and the system sensitivity, the only parameters left to tune are the noise power spectral density of the *U*_lcpd_ transitions and sideband observations. Because the latter is easily determined from a power spectrum near the sidebands, the controller performance can be tuned in practice using the transition noise only. Independent of the chosen parameters, the feedback loop will be stable thanks to the Kalman filter structure.

## Supporting Information

File 1 Detailed derivations of the effective forces and the state-space KFM controller.
