# Supplementary material for: Kelvin probe force microscopy for local characterisation of active nanoelectronic devices
Source: Beilstein J Nanotechnol. 2015 Nov 23;6:2193–206. doi: 10.3762/bjnano.6.225 (PMC4685916; doi:10.3762/bjnano.6.225)
Supplement: File 1 — Detailed derivations of the effective forces and the state-space KFM controller. [file Beilstein_J_Nanotechnol-06-2193-s001.pdf]

Supporting Information
for
"Kelvin probe force microscopy for local characterisation of active nanoelectronic devices"

Tino Wagner[1], Hannes Beyer[1], Patrick Reissner[1], Philipp Mensch[2], Heike Riel[2], Bernd Gotsmann[2], and Andreas Stemmer[1]

[1]Nanotechnology Group, ETH Zürich, Säumerstrasse 4, 8803 Rüschlikon, Switzerland
[2]IBM Research — Zurich, Säumerstrasse 4, 8803 Rüschlikon, Switzerland

1 Effective electrostatic tip–sample forces and force gradients

In Figure 1 of the main text, we showed the effective C' and C'' as a function of the tip–sample distance, calculated for different amplitudes from an analytical approximation of the electrostatic force. We use the following expressions for the electrostatic force, derived by Hudlet *et al.* [1], for the spherical apex and cone

$$F_{\text{apex}}(z) = -\pi\epsilon_0\, U_{\text{ts}}^2 \frac{R\tilde{R}}{z(z+\tilde{R})} = -\pi\epsilon_0 R\, U_{\text{ts}}^2 \left(\frac{1}{z} - \frac{1}{z+\tilde{R}}\right) \tag{1}$$

$$F_{\text{cone}}(z) = -\pi\epsilon_0 k^2\, U_{\text{ts}}^2 \left[\ln\frac{H}{z+\tilde{R}} - 1 + \frac{R\cos^2\theta_0/\sin\theta_0}{z+\tilde{R}}\right], \tag{2}$$

where $k^2 = (\ln\tan\theta_0/2)^{-2}$, $\tilde{R} = R(1-\sin\theta_0)$, R is the radius of the apex, θ_0 is the half opening angle of the cone, H is the total height of the tip formed by apex and cone, and z is the distance of the apex to the sample plane. In addition, we consider the contribution of the cantilever as a plate capacitor (effective area A) with a separation of $z+H$,

$$F_{\text{lever}}(z) = -\frac{1}{2}\frac{\epsilon_0 A}{(z+H)^2}\, U_{\text{ts}}^2. \tag{3}$$

The forces as given above include only terms following either a power law or logarithmic dependence on the distance. In these cases, the integrals for the effective force and force gradient during an oscillation $z = d + A(1+\cos\omega t)$, can be solved as follows. For an inverse power law, $F_{\text{ts}}(z) = -C(z+h)^{-n}$, using eqs. (2) and (3) in the main text, we find

$$\langle F_{\text{ts}}\rangle(d) = -C\frac{\sqrt{2}}{\pi}(d+h)^{-n+\frac{1}{2}}A^{-\frac{1}{2}}\int_0^{\sqrt{\frac{2A}{d+h}}} \mathrm{d}y\,(1+y^2)^{-n}\left(1-\frac{d+h}{2A}y^2\right)^{-\frac{1}{2}} \tag{4}$$

$$= -C(d+h)^{-n}{}_2F_1\left(\frac{1}{2}, n; 1; -\frac{2A}{d+h}\right) \tag{5}$$

$$= -C(d+A+h)^{-n}{}_2F_1\left(\frac{n}{2}, \frac{n+1}{2}; 1; \frac{A^2}{(d+A+h)^2}\right), \text{ and} \tag{6}$$

$$k_{\rm ts}(d) = C\frac{\sqrt{2}}{\pi}(d+h)^{-n+\frac{1}{2}}A^{-\frac{3}{2}}4n\int_0^{\sqrt{\frac{2A}{d+h}}}\mathrm{d}y\,y^2(1+y^2)^{-n-1}\left(1-\frac{d+h}{2A}y^2\right)^{\frac{1}{2}} \tag{7}$$

$$= nC(d+h)^{-n-1}{}_2F_1\left(\frac{3}{2}, n+1; 3; -\frac{2A}{d+h}\right) \tag{8}$$

$$= nC(d+A+h)^{-n-1}{}_2F_1\left(\frac{n+1}{2}, \frac{n+2}{2}; 2; \frac{A^2}{(d+A+h)^2}\right), \tag{9}$$

where the definite integrals are expressed[1] in terms of Gauss' hypergeometric function, ${}_2F_1(a, b; c; z)$. For large amplitudes, $d + h \ll 2A$, the integrals above may be approximated in terms of the gamma function:[2]

$$\langle F_{\rm ts}\rangle(d) \approx -\frac{C}{\sqrt{2\pi}}(d+h)^{-n+\frac{1}{2}}A^{-\frac{1}{2}}\frac{\Gamma(n-\frac{1}{2})}{\Gamma(n)} \tag{10}$$

$$k_{\rm ts}(d) \approx C\sqrt{\frac{2}{\pi}}(d+h)^{-n+\frac{1}{2}}A^{-\frac{3}{2}}\frac{\Gamma(n-\frac{1}{2})}{\Gamma(n)}. \tag{11}$$

An equivalent expression for the force gradient (eq. (11)) is also given by Giessibl [4], and motivates the normalised frequency shift in frequency modulated AFM, $\gamma = kA^{3/2}\Delta f/f_0$. Similarly, a normalised force would be $\propto \sqrt{A}$.

With the wide range of values for $(d + h)/2A$, as required for Figure 1 in the main text, above approximations do not necessarily hold. For an estimate of the error, see Figure S1. We therefore evaluate ${}_2F_1$ numerically for exact results.

For a logarithmic law, $F_{\rm ts}(z) = C \ln(z + h)$, we get

$$\langle F_{\rm ts}\rangle(d) = C\ln(d+h) + 2C\ln\left[\frac{1}{2}\left(1+\sqrt{1+\frac{2A}{d+h}}\right)\right] \tag{12}$$

$$k_{\rm ts}(d) = C(d+h)^{-1}{}_2F_1\left(\frac{3}{2}, 1; 3; -\frac{2A}{d+h}\right) \tag{13}$$

$$= \frac{2C}{A}\left[1+\frac{d+h}{A}\left(1-\sqrt{1+\frac{2A}{d+h}}\right)\right]. \tag{14}$$

The total electrostatic force is $F_{\rm el} = F_{\rm apex} + F_{\rm cone} + F_{\rm lever}$. Since $F_{\rm el} = C' U_{\rm ts}^2/2$, the capacitance gradients C' and C'' follow straightforwardly from the expressions of the electrostatic forces and force gradients, respectively.

2 The frequency modulation limit

With $\tilde{z}(\omega) = \sqrt{2\pi}\sum_{n=-\infty}^{\infty}\hat{z}_n\delta(\omega_{\rm d} + n\omega_{\rm m} - \omega)$, eq. (4) in the main text can be written as a recurrence relation:

$$G^{-1}(\omega_{\rm d} + n\omega_{\rm m})\,\hat{z}_n = \hat{a}\delta_{n0} + \frac{\hat{k}_{\rm ts}}{2k}\left[\hat{z}_{n-1} + \hat{z}_{n+1}\right]. \tag{15}$$

---

[1] Substitute $t = (d + h)y^2/2A$ and use the integral formula [2]

$${}_2F_1(a,b;c;z) = \frac{\Gamma(c)}{\Gamma(b)\Gamma(c-b)}\int_0^1 \mathrm{d}t\, t^{b-1}(1-t)^{c-b-1}(1-zt)^{-a}.$$

Equations (6) and (9) are obtained by applying the quadratic transformation formula 15.3.16 in Abramowitz & Stegun [2]. The latter step returns the argument of ${}_2F_1$ into the unit circle, such that an evaluation is possible in terms of its series definition

$${}_2F_1(a,b;c;z) = \sum_{k=0}^{\infty}\frac{(a)_k(b)_k}{(c)_k}\frac{z^k}{k!} \quad \text{for} \quad |z| < 1.$$

[2] Let $(d + h)/A \to 0$ and use $\int_0^1 \mathrm{d}x\, x^a(1 - x)^b = \frac{\Gamma(a+1)\Gamma(b+1)}{\Gamma(a+b+2)}$ [3].

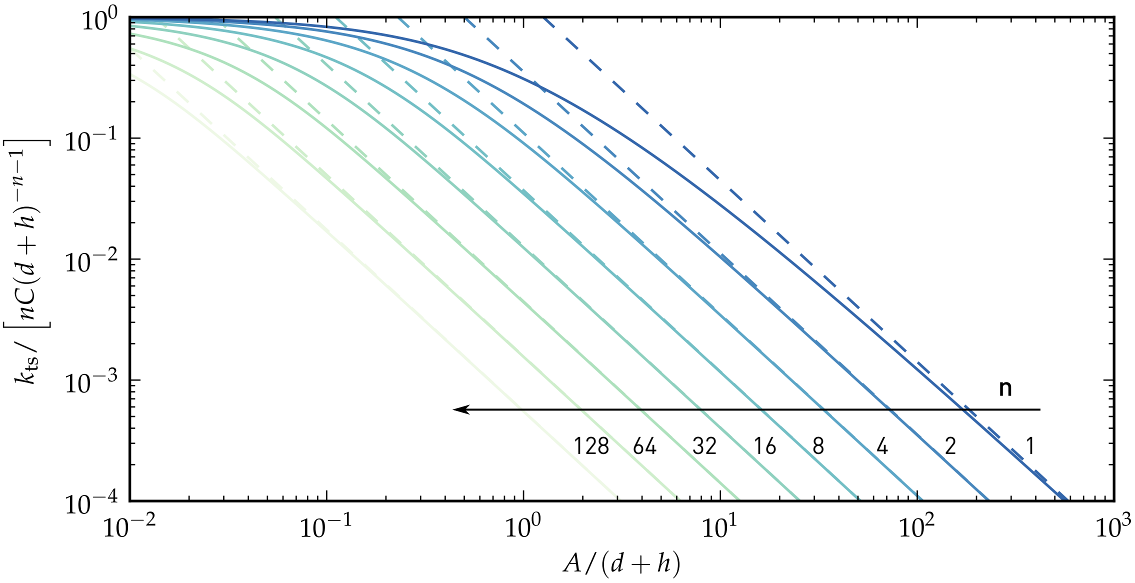

Figure S1: Effective force gradient for an inverse power law, $F_{\text{ts}} = -C(z+h)^{-n}$, exact (solid, eq. (8)) and approximated for large amplitudes (dashed, eq. (11)) for different powers n. k_{ts} is normalised to the corresponding expression at vanishing oscillation amplitudes.

For $\omega_{\text{d}} = \omega_0$, $\omega_{\text{m}} \ll \omega_0$, $Q \gg 1$, and $n \neq 0$, the above relation simplifies to

$$\hat{z}_{n+1} + \hat{z}_{n-1} = 2n \frac{\omega_{\text{m}}}{\Delta\hat{\omega}} \hat{z}_n, \tag{16}$$

which resembles the recurrence relation $J_{n+1} + J_{n-1} = (2n/\beta)\, J_n$ [2] for the Bessel functions of the first kind, $J_n(\beta = \Delta\hat{\omega}/\omega_{\text{m}})$. In this limit, the sidebands are the same as in frequency modulation, and β is the modulation index.

3 A state-space controller for FM-KFM

In the main text, we have shown that the sideband dynamics after lock-in detection can be modelled as a n-th order critically damped low-pass filter with the transfer function $G(s) = K(1+\tau s)^{-n}$. In state space, the same system can be written as $\dot{\mathbf{x}}^\circ = \mathbf{A}^\circ \mathbf{x}^\circ + \mathbf{B}^\circ u^\circ$ and $y = \mathbf{C}^\circ \mathbf{x}^\circ$ with the input $u^\circ = U_{\text{dc}} - U_{\text{lcpd}}$ and the system matrices

$$\mathbf{A}^\circ = \frac{1}{\tau} \begin{pmatrix} -1 & 0 & \cdots & 0 \\ 1 & -1 & \cdots & 0 \\ \vdots & & \ddots & 0 \\ 0 & \cdots & 1 & -1 \end{pmatrix} \in \mathbb{R}^{n\times n}, \tag{17}$$

$$\mathbf{B}^\circ = \begin{pmatrix} \frac{1}{\tau} & 0 & \cdots & 0 \end{pmatrix}^\intercal \in \mathbb{R}^{n\times 1}, \text{ and} \tag{18}$$

$$\mathbf{C}^\circ = \begin{pmatrix} 0 & \cdots & 0 & K \end{pmatrix} \in \mathbb{R}^{1\times n}. \tag{19}$$

In this formulation, each element of the state vector $\mathbf{x}^\circ$ describes the input signal after each filter stage. By rewriting the system to include the surface potential as the *hidden* state $x_{n+1} =$

U_{lcpd}, the new system is $\dot{\mathbf{x}} = \mathbf{A}\mathbf{x} + \mathbf{B}u$, $y = \mathbf{C}\mathbf{x}$, with the input $u = U_{\mathrm{dc}}$ and the system matrices

$$\mathbf{A} = \begin{pmatrix} & -\frac{1}{\tau} \\ \mathbf{A}^{\circ} & \vdots \\ & 0 \\ \mathbf{0} & 0 \end{pmatrix} \in \mathbb{R}^{n+1\times n+1}, \tag{20}$$

$$\mathbf{B} = \begin{pmatrix} \mathbf{B}^{\circ\intercal} & 0 \end{pmatrix}^{\intercal} \in \mathbb{R}^{n+1\times 1}, \text{ and} \tag{21}$$

$$\mathbf{C} = \begin{pmatrix} \mathbf{C}^{\circ} & 0 \end{pmatrix} \in \mathbb{R}^{1\times n+1}. \tag{22}$$

The task of finding the surface potential is therefore reduced to estimating the hidden state, U_{lcpd}, from the observation y.

We include measurement noise in the observations y and the uncertainty of the surface potential as white noise sources. Therefore

$$\dot{\mathbf{x}} = \mathbf{A}\mathbf{x} + \mathbf{B}u + v \tag{23}$$

$$y = \mathbf{C}\mathbf{x} + w, \tag{24}$$

where v and w are white noise processes with known spectral densities and covariance matrices $\mathbf{V}$ and $\mathbf{W}$, respectively.

An estimate of the state, $\hat{\mathbf{x}}$, is derived from the observations, y, using an observer: It uses the information about the dynamics of the state, modelled within the matrices $\mathbf{A}$ and $\mathbf{B}$, and adds to it a scaled version of the residual, $y - \mathbf{C}\hat{\mathbf{x}}$:

$$\dot{\hat{\mathbf{x}}} = \mathbf{A}\hat{\mathbf{x}} + \mathbf{B}u + \mathbf{L}(y - \mathbf{C}\hat{\mathbf{x}}), \tag{25}$$

where $\mathbf{L}$ is the filter gain. That is, knowledge about the system is used to derive a state estimate from measurements.

The optimum observer minimises the sum of the squared residuals over time. Given that in our case the system is perturbed by white noise, the optimum observer is the Kalman filter[5] with

$$\mathbf{L} = \mathbf{P}\mathbf{C}^{\intercal}\mathbf{W}^{-1}, \tag{26}$$

where $\mathbf{P}$ is the covariance matrix of the state estimate, which is found from the solution of the differential Riccati equation[6]

$$\dot{\mathbf{P}} = \mathbf{A}\mathbf{P} + \mathbf{P}\mathbf{A}^{\intercal} - \mathbf{P}\mathbf{C}^{\intercal}\mathbf{W}^{-1}\mathbf{C}\mathbf{P} + \mathbf{V}. \tag{27}$$

With $\dot{\mathbf{P}} = 0$, a steady-state solution can be found, allowing one to derive steady-state transfer functions and simplified state update equations.

References

1. Hudlet, S., Saint Jean, M., Guthmann, C. & Berger, J. Evaluation of the capacitive force between an atomic force microscopy tip and a metallic surface. *Eur. Phys. J. B* **2,** 5 (1998).
2. Abramowitz, M. & Stegun, I. A. *Handbook of Mathematical Functions* (Dover New York, 1972).
3. Bronstein, I. N., Semendjaew, K. A., Grosche, G., Ziegler, V. & Ziegler, D. *Springer-Taschenbuch der Mathematik* (ed Zeidler, E.) (Springer Fachmedien Wiesbaden, Wiesbaden, 2013).
4. Giessibl, F. J. Forces and frequency shifts in atomic-resolution dynamic-force microscopy. *Physical Review B (Condensed Matter)* **56,** 90128–16015 (1997).
5. Kalman, R. E. & Bucy, R. S. New Results in Linear Filtering and Prediction Theory. *J. Basic Eng* **83,** 95–108 (1961).
6. Friedland, B. *Control System Design: An Introduction to State-Space Methods* (Dover, 2005).
